# Neurorrhaphy in Presence of Polyethylene Glycol Enables Immediate Electrophysiological Conduction in Porcine Model of Facial Nerve Injury

**DOI:** 10.3389/fsurg.2022.811544

**Published:** 2022-03-07

**Authors:** Dmitriy Petrov, Justin C. Burrell, Kevin D. Browne, Franco A. Laimo, Sanford E. Roberts, Zarina S. Ali, D. Kacy Cullen

**Affiliations:** ^1^Center for Brain Injury and Repair, Department of Neurosurgery, Perelman School of Medicine, University of Pennsylvania, Philadelphia, PA, United States; ^2^Center for Neurotrauma, Neurodegeneration and Restoration, Corporal Michael J. Crescenz Veterans Affairs Medical Center, Philadelphia, PA, United States; ^3^Department of Bioengineering, School of Engineering and Applied Science, University of Pennsylvania, Philadelphia, PA, United States

**Keywords:** swine, facial nerve injuries, peripheral nerve injuries, facial paralysis, axons, nerve repair, polyethylene glycol, nerve fusion

## Abstract

Facial nerve trauma often leads to disfiguring facial muscle paralysis. Despite several promising advancements, facial nerve repair procedures often do not lead to complete functional recovery. Development of novel repair strategies requires testing in relevant preclinical models that replicate key clinical features. Several studies have reported that fusogens, such as polyethylene glycol (PEG), can improve functional recovery by enabling immediate reconnection of injured axons; however, these findings have yet to be demonstrated in a large animal model. We first describe a porcine model of facial nerve injury and repair, including the relevant anatomy, surgical approach, and naive nerve morphometry. Next, we report positive findings from a proof-of-concept experiment testing whether a neurorrhaphy performed in conjunction with a PEG solution maintained electrophysiological nerve conduction at an acute time point in a large animal model. The buccal branch of the facial nerve was transected and then immediately repaired by direct anastomosis and PEG application. Immediate electrical conduction was recorded in the PEG-fused nerves (*n* = 9/9), whereas no signal was obtained in a control cohort lacking calcium chelating agent in one step (*n* = 0/3) and in the no PEG control group (*n* = 0/5). Nerve histology revealed putative-fused axons across the repair site, whereas no positive signal was observed in the controls. Rapid electrophysiological recovery following nerve fusion in a highly translatable porcine model of nerve injury supports previous studies suggesting neurorrhaphy supplemented with PEG may be a promising strategy for severe nerve injury. While acute PEG-mediated axon conduction is promising, additional work is necessary to determine if physical axon fusion occurs and the longer-term fate of distal axon segments as related to functional recovery.

## Highlights

- Characterized anatomy, surgical approach, and morphometry of facial nerve in Yorkshire swine.
- Demonstrated immediate electrophysiological conduction after direct facial nerve repair following application of polyethylene glycol (PEG).
- Established platform to optimize future mechanistic studies investigating fusogen-mediated axon continuity.
- Porcine facial nerve injury model has utility for the optimization of next-generation neurosurgical repair strategies.

## Introduction

Facial nerve palsy resulting from facial nerve injury is a devastating, disfiguring condition, diagnosed in ~20 in 100,000 patients annually (1). Muscle paralysis may occur from trauma to the facial nerve and resultant denervation of the facial muscle (2). Moreover, severe nerve trauma resulting from major facial injury or tumor resection leads to prolonged denervation and the subsequent breakdown of the pro-regenerative environment and motor end plates necessary for ensuring meaningful recovery (3, 4). After the nerve injury, transected axons rapidly seal to prevent further damage *via* calcium influx and calcium-mediated vesicular formation (5). After 3–7 days, the disconnected distal axonal segments undergo fragmentation and eventually myelin clearance (6). Fusogens, such as polyethylene glycol (PEG), are a promising approach for nerve repair that are posited to immediately restore axonal continuity, and thereby preventing Wallerian degeneration (7–10). After administering calcium-free hypotonic saline to prevent calcium influx and an antioxidant to prevent free-radical formation as well as vesicular formation, fusogens can be applied which may rapidly restore the connection between the unsealed proximal axons in close opposition to unsealed distal axons. Therefore, it has been suggested that the successful execution of the fusogen protocol facilitates the reconnection of otherwise transected axonal membranes, allowing for immediate electrophysiological connectivity.

Although PEG fusion is an exciting area in nerve repair, it remains unclear whether physical axon fusion occurs and directly impacts the fate of distal axon segments. While several studies have shown that PEG fusion may enable the immediate electrophysiological reconnection in rats, further testing in large animal models is necessary since the extent that neurobiological mechanisms involved in injury and regeneration are conserved across species remains unclear (11, 12). Here, we present a proof-of-concept study demonstrating PEG-mediated nerve fusion in a porcine facial nerve model. This porcine facial nerve injury model offers significant utility as a translational pre-clinical model and may provide an ideal platform for testing of next-generation surgical techniques and advanced therapies for nerve repair.

## Methods

All procedures were approved by the University of Pennsylvania's Institutional Animal Care and Use Committee (Protocol #805788) and adhered to the guidelines set forth in the NIH Public Health Service Policy on Humane Care and Use of Laboratory Animals (2015).

### Surgical Preparation and Operating Technique

Female Yorkshire pigs (3 months old, 25–35 kg; Animal Biotech Industries) were used to study the efficacy of nerve fusion immediately after repair [*n* = 12 nerves (6 swine) with PEG application and *n* = 5 nerves (3 swine) without PEG application]. Anesthesia, preoperative, and postoperative management were completed as described previously (13). Each animal in the study received bilateral facial nerve repairs, therefore, unless otherwise specified, sample size represents the number of nerves per group.

A 4.0 cm incision was made from the mandible toward the lateral commissure, ~1 cm inferior and parallel to the zygoma. The subcutaneous fat and facial tissue were dissected to expose the trunk of the facial nerve coursing on the masseter muscle. Smaller nerves innervating the eyelid were cut to isolate the buccal branch of the facial nerve innervating the upper lip and snout. After exposure, the facial nerve was irrigated with calcium-free PlasmaLyte-A supplemented with a calcium chelating agent (0.5 mM egtazic acid; EGTA). The nerve was sharply transected and bathed with additional calcium-free PlasmaLyte-A.

A tension-less end-to-end nerve repair was completed using four 8-0 prolene sutures. In this study, we tested two different PEG application protocols. In the first PEG protocol, after applying calcium-free Plasmalyte-A + EGTA, hypotonic 1% methylene blue (MB) solution was diluted in calcium-free PlasmaLyte-A supplemented with EGTA, which was applied immediately before tightening the sutures followed by administration of PEG (molecular weight: 3,350). In the second PEG protocol, hypotonic 1% MB was diluted in diH_2_O without calcium chelating agent (EGTA). Lactated ringer's solution was applied to wash away excess PEG. Negative control nerves were washed with calcium-free PlasmaLyte-A with EGTA and hypotonic 1% MB diluted in calcium-free Plasmalyte-A with EGTA before suturing, and lactated ringer's solution was then applied similar to the PEG protocols. Electrophysiological recordings were performed immediately before and after repair to evaluate acute functional recovery.

### Facial Nerve Electrophysiology Recordings

Compound nerve action potentials (CNAPs) were obtained following proximal nerve stimulation from a distal recording electrode (amplitude: 0–10 mA, duration: 0.1 ms, bandpass filter: 10–10,000 Hz + 60 Hz notch filter, 1,000× gain). The stimulating and recording electrodes were positioned 0.5 cm caudal and rostral to the repair site, respectively. The ground electrode was inserted into subcutaneous tissue halfway between the electrodes. CNAP amplitude was measured as the peak-to-peak from the positive deflection to negative deflection. A binary score for successful evoked muscle response was recorded by stimulating the proximal nerve after repair. A 3 × 2 contingency table was generated for the three cohorts and the two outcomes (successful or failed evoked response). We were unable to run a traditional chi-square comparison since the outcome for two of the groups were zero. Therefore, data were compared using Fisher's exact test between individual groups (Graph Pad Prism Version 8, La Jolla, California).

### Euthanasia, Tissue Processing, Histology, and Microscopy

Animals were euthanized immediately following the acute electrophysiological assessment by transcardial perfusion with heparinized saline followed by 10% neutral-buffered formalin. Repaired nerves were harvested for morphometric analyses spanning 5 mm proximal and distal to the suture site. Nerves were fixed in 10% neutral-buffered formalin at 4°C overnight. For longitudinal histological assessment of the repair zone, nerves were placed in 30% sucrose overnight and then subsequently embedded in optimal cutting media, and frozen in dry ice/isopentane. The nerve was sectioned longitudinally at a thickness of 20 μm and mounted on glass slides for staining. Sections were rinsed in PBS (3 × 5 min) and blocked for 1 h in blocking solution (PBS with 4% normal horse serum and 0.3% Triton X-100). Axons were labeled with mouse anti-SMI31/32 (1:1000, BioLegend Cat# 801601 and BioLegend Cat# 701601) diluted in blocking solution following overnight incubation at 4°C. Slides were rinsed in PBS (3 × 5 min) and donkey anti-mouse 488 diluted in blocking solution (1:500) was applied for 2 h at room temperature. Sections were then rinsed in PBS (3 × 5 min), mounted with Fluoromount-G® (Southern Biotech Cat#0100-01) and coverslipped.

Fluorescent images were obtained with a Nikon A1R confocal microscope (1,024 × 1,024 pixels) with a 10× air objective or 60× oil objective using Nikon NIS-Elements AR 3.1.0 (Nikon Instruments, Tokyo, Japan).

## Results

### Surgical Exposure of the Facial Nerve

**Figure 1:**
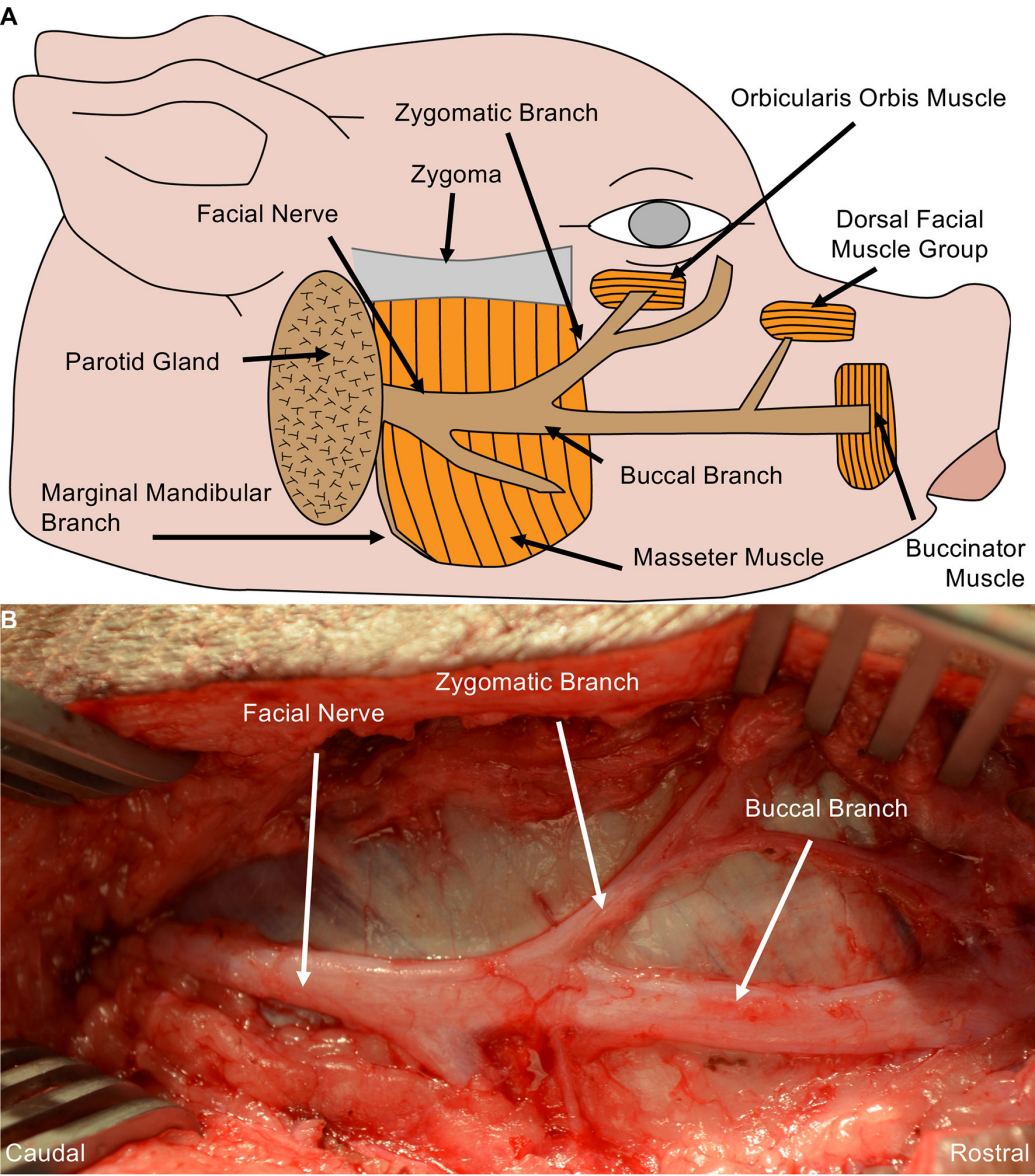
Porcine Facial Nerve Anatomy. **(A)** Drawing denoting relevant facial nerve anatomy. **(B)** Surgical perspective of the facial nerve and its distal branches were performed in Yorkshire cadavers. A linear incision was made from the mandible towards the lateral commissure, approximately 1 cm inferior and parallel to the zygoma. Blunt dissection of the subcutaneous fat and facial exposed the trunk of the facial nerve coursing over the masseter muscle. The facial nerve and its branches were completely dissected from the surrounding tissue and followed to each muscle end-target. The marginal mandibular branch of the facial nerve originates from the ventral aspect of the facial nerve, coursing under the mandible to the rostral muscle end target(s) (partially shown here). Approximately 6–7 cm of the buccal branch was exposed before innervating the muscle end target.

Detailed anatomic dissections were carried out in Yorkshire cadavers to gain an understanding of the peripheral nerve origins, trajectories, and branching patterns. Based on those findings, we determined that making a linear incision ~1 cm inferior and parallel to the zygoma and extending from the base of the pinna toward the lateral commissure provided optimal access to the complete structure of the facial nerve. Subsequent to incision, the subcutaneous tissue and fascia were dissected to expose the trunk of the facial nerve coursing on the masseter muscle. The facial nerve has a large ellipsoidal diameter and branched into the mandibular nerve, zygomatic nerve, and the buccal nerve. The mandibular nerve coursed inferiorly toward the jaw, zygomatic nerve superiorly toward the eyelid, and the buccal nerve rostrally toward the snout (Figure 1).

### Structural Findings in Naive Facial Nerves

**Figure 2:**
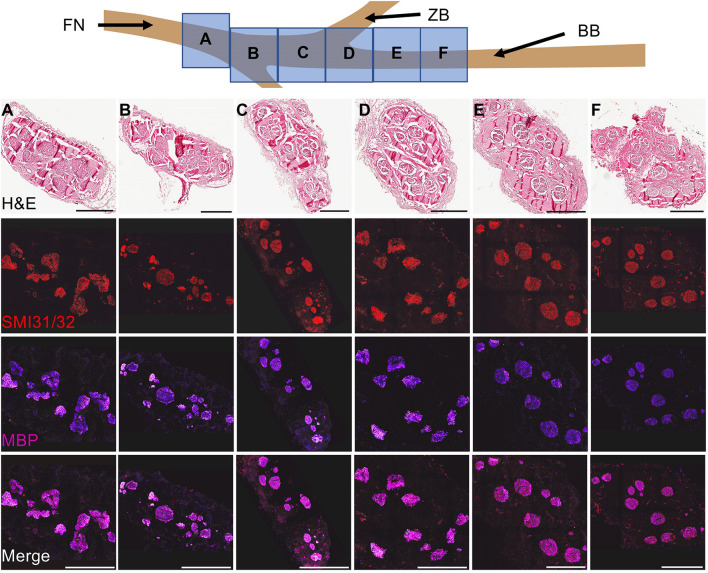
Cross-Sectional Naive Facial Nerve Histological Characterization. To characterize the facial nerve fascicular architecture, the facial nerve and its branches were harvested, fixed, and subsequently blocked every centimeter starting 1 cm before the bifurcation point of the ventral and dorsal segments of the buccal branch. Cross-sectioned nerves (8 μm thick) were stained to identify bundles of axons (SMI31/32, red) and myelin (myelin basic protein, purple). Naive facial nerve histological assessment of the axon morphometry revealed large and small myelinated fibers organized in a polyfascicular pattern. The fascicular architecture changed as the nerve coursed caudal to rostral. At the caudal end, a dense fascicular structure was found relative to the total nerve diameter. During the rostral course of the facial nerve, the total nerve area flattened and individual branches were formed within separate epineurium. Notably, compared to the caudal end of the facial nerve, the buccal branch had a relatively sparse fascicular area relative to the total nerve. Abbreviations: FN- Facial nerve, ZB- Zygomatic branch. Scale: 500 μm.

Naive facial nerve histological assessment of the axon morphometry revealed large and small myelinated fibers organized in a polyfascicular pattern. At the caudal end, the facial nerve had a denser fascicular structure relative to the total nerve diameter. As the nerve coursed rostrally, the facial nerve became flatter and epineurium began to diverge into the individual branches. The buccal branch was identified as the main branch innervating the dorsal muscle group (Figure 2).

### Electrophysiological Response Immediately After Facial Nerve Neurorrhaphy in Presence of PEG Solutions

**Figure 3:**
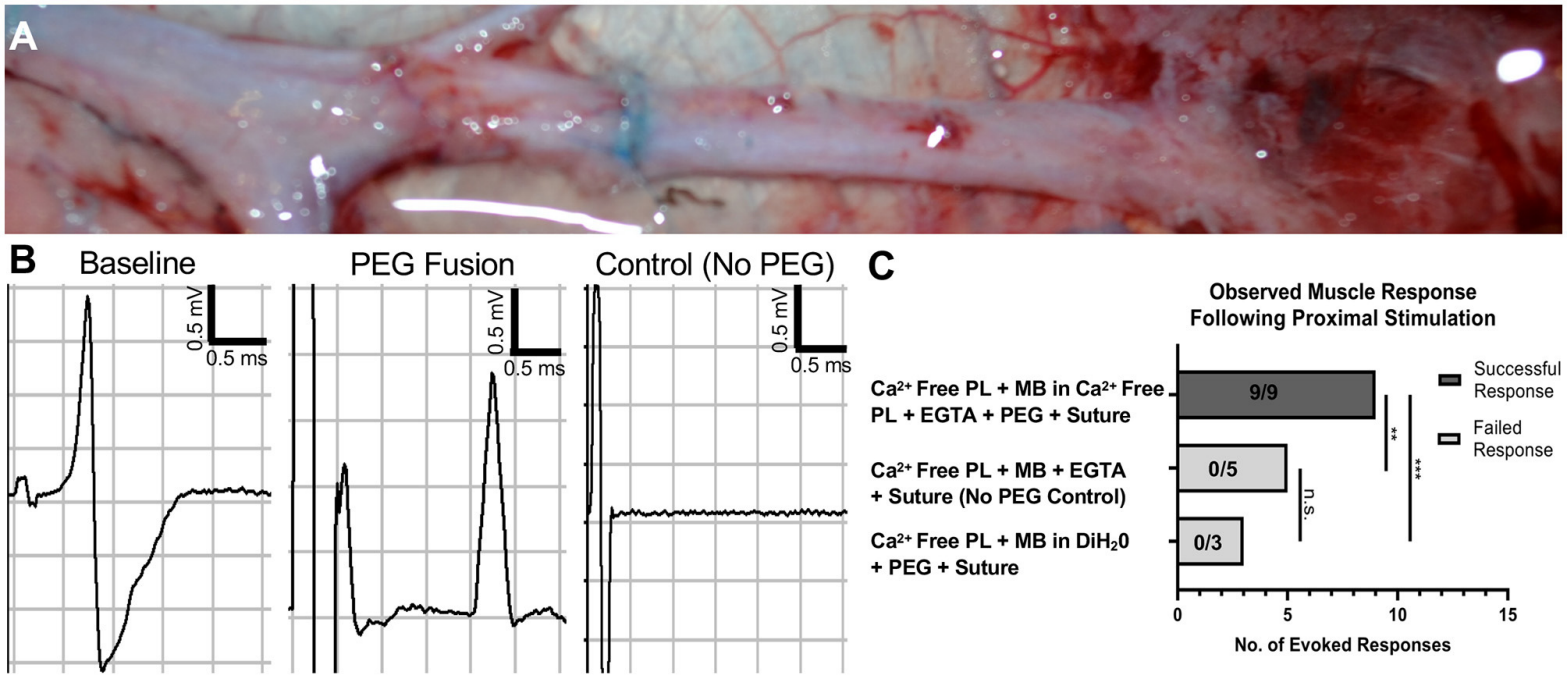
Restoration of axon conduction immediately following PEG-mediated direct facial nerve repair. **(A)** Intraoperative photo immediately following PEG fusion. Briefly, the nerve was transected in hypotonic saline, and epineurial sutures were placed to approximate the direct nerve repair. Methylene blue was then applied to the nerve and enters the injury site, which can be seen in **(A)**. Nerve fusion is achieved by applying PEG to the repair site as the direct repair is completed by tying the epineurial sutures. **(B)** Compound nerve action potentials (CNAP) were performed immediately before and after repair. Immediate electrical connectivity with a robust waveform was noted following successful PEG fusion. No response was observed in control animals lacking PEG application. **(C)** An evoked muscle response was seen in 9/9 in the PEG fusion cohort treated with calcium-free PlasmaLyte-A + EGTA, methylene blue in calcium-free PlasmaLyte-A + EGTA, PEG, and then nerve suture. No evoked response was found in the cohort treated with calcium-free PlasmaLyte-A + EGTA, methylene blue in deionized water, PEG, and then nerve suture (0/3). Additionally, no response was observed in the negative control cohort lacking PEG application (0/5) (**p < 0.01, ***p < 0.001, n.s. no significance). PEG, Polyethylene Glycol; PL, Calcium-free PlasmaLyte-A; MB, methylene blue; DiH_2_O, deionized water; EGTA, Egtazic acid.

Facial nerve neurorrhaphy was completed after following two different PEG application protocols as well as a negative control protocol. CNAP electrophysiological assessment was performed immediately before and after repair. Successful evoked muscle response was found in 9/9 nerves following neurorrhaphy after applying PlasmaLyte-A supplemented with EGTA, 1% MB diluted in PlasmaLyte-A supplemented with EGTA, and PEG compared to 0/5 nerves in the negative control cohort (*p* < 0.001) (Figure 3). Interestingly, there was no evoked response in 0/3 nerves after following a similar protocol if MB was diluted in diH_2_O lacking EGTA (*p* < 0.001 compared to the first PEG fusion protocol). CNAP waveforms obtained following the first PEG protocol were qualitatively different than naive traces pre-repair, whereas no signal was detected in the negative control, suggesting the electrophysiological activity was physiological rather than simply current spreading across the suture site.

### Histological Findings Immediately After Facial Nerve Neurorrhaphy in Presence of PEG Solutions

**Figure 4:**
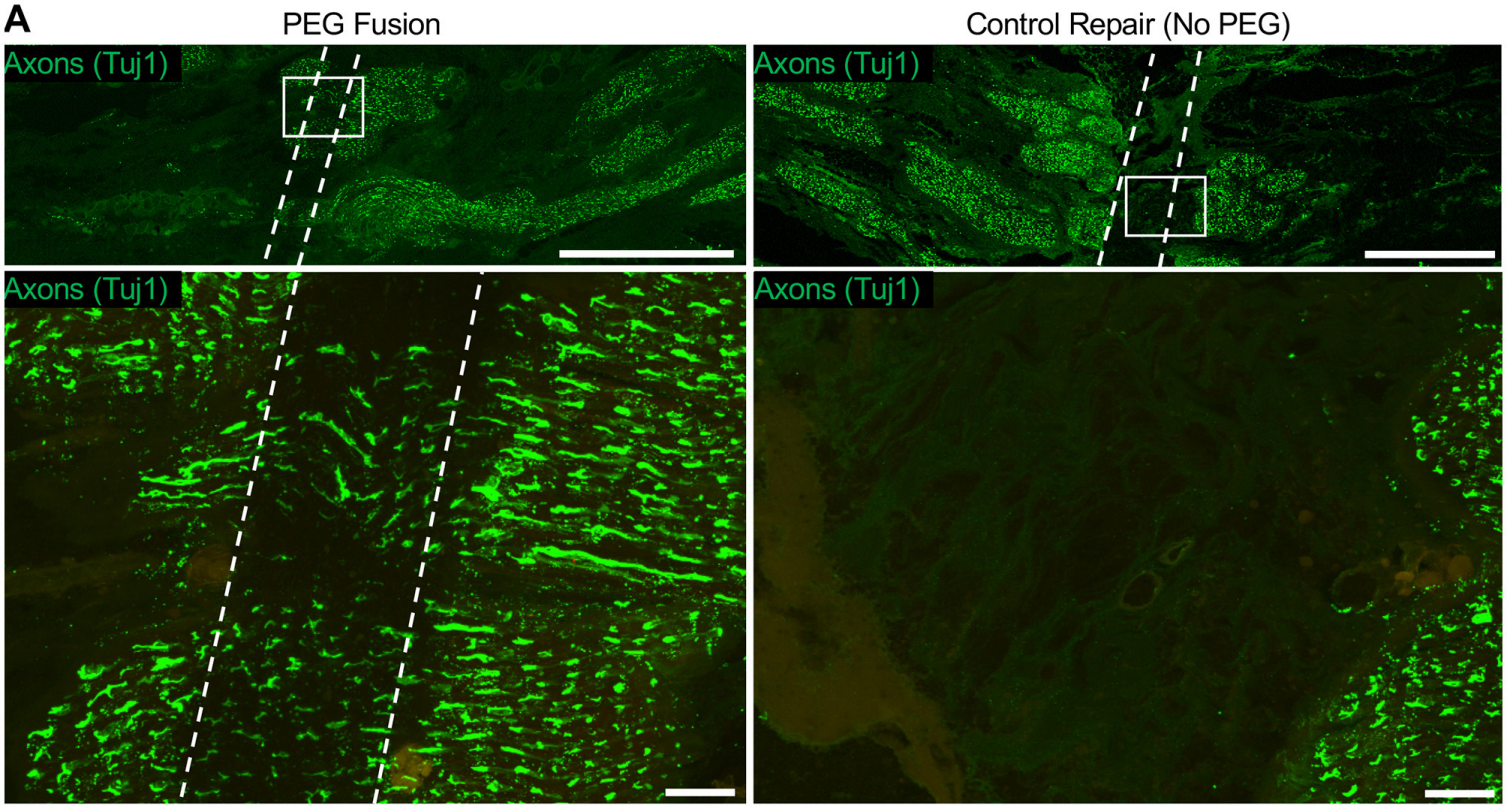
Putative axon continuity immediately following direct facial nerve repair. **(A)** Nerves were sectioned at the repair site longitudinally and stained for axons (SMI31/32, green). At low magnification, the repair site can be clearly seen as the region lacking axon connectivity between the facial nerve stumps. High magnification revealed axons spanning the repair site following neurorrhaphy + PEG, whereas no axons were seen in the control nerve (neurorrhaphy without PEG).

Whole-mount tissue and longitudinal sections were stained with SMI31/32 to identify axons. Axons rostral and caudal to the repair site were readily observed immediately after neurorrhaphy, as expected (Figure 4). Histological assessment of the PEG cohort treated with MB diluted in PlasmaLyte-A with EGTA revealed that no axons were found in the repair site if there was no immediate electrophysiological recovery after repair. At high magnification, axons were observed with clear fascicular alignment between the caudal and rostral nerve stumps, indicating that careful approximation is likely important.

## Discussion

Severe peripheral nerve injuries are surprisingly common following motor vehicle accidents, sports-related injuries, and iatrogenic injuries (14–16). Recovery from peripheral nerve injury can be arduous, with long recovery times and often poor return of function. Much of this can be attributed to slow axonal regrowth, long regenerative distances, Wallerian degeneration, and prolonged denervation leading to muscle atrophy (6). PEG has been well-described as an artificial fusogen in quickly reconnecting severed ends of giant invertebrate axons and rodents (7–10, 17–25). Recent findings in rodent models have suggested that PEG fusion may mitigate the harmful effects of prolonged denervation by preventing Wallerian degeneration and rapidly restoring the electrophysiological connection of some axons.

In a proof-of-concept study, we tested the feasibility of neurorrhaphy with PEG fusion protocols in a porcine facial nerve injury model. We found direct facial nerve anastomosis with PEG fusion protocols immediately enabled a measurable CNAP across the suture site that resulted in an evoked snout twitch similar to previous studies. However, acute electrophysiological conduction alone does not definitively show immediate axon fusion (i.e., restored continuity). Indeed, post-mortem immunohistochemistry and microscopic analyses revealed the presence of axons spanning the defect in the cohort of animals with successful evoked responses following the PEG fusion protocol with MB diluted in PlasmaLyte-A with EGTA, suggesting putative fusion at the time of stimulation. Future studies are necessary that utilize additional techniques such as tract tracing with tissue clearing and/or electron microscopy to demonstrate the potential efficacy of actual axonal fusion. While the present study is limited to an acute time point, the main goal was to demonstrate whether PEG-mediated electrophysiological capacity was maintained in a large animal model of nerve injury. Future work will be required to establish the longer-term fate of the transected axons; in particular whether the distal segments undergo Wallerian degeneration or if a subset that may have fused to proximal segments are spared.

Several protocols reporting successful PEG fusion have been previously published. However, the main components of the protocol are: (1) Transect the nerve in calcium-free hypotonic saline (e.g., PlasmaLyte-A) to prevent calcium-mediated vesicular budding; (2) Apply an antioxidant (e.g., MB) to prevent the free radical formation and keep the axonal ends open; (3) Closely oppose nerve using sutures; (4) Apply PEG at the repair site before finishing suturing to allow for fusion of the open axonal ends; (5) Wash the surgical field thoroughly with calcium-containing isotonic solution (e.g., lactated ringers) to seal any unfused axons. Slight modifications have been published, such as applying hypotonic calcium-free saline with a calcium chelator (EGTA) and MB diluted in calcium-free saline (20). One potential advantage of using calcium chelating agent is that it has been reported to improve the efficiency of PEG fusion in large myelinated earthworm axons (25). However, successful PEG fusion has been reported in rodents after transecting the nerve in calcium-free saline followed by MB diluted in distilled water (diH_2_O) (5, 7, 31). In this study, we tested two potential PEG fusion protocols with slightly different MB dilutions. In the first protocol, MB was diluted in calcium-free saline (PlasmaLyte-A) supplemented with a calcium chelating agent (EGTA). In the second protocol, MB was diluted in diH_2_O lacking a calcium chelator. While immediate electrical connectivity was restored after completing the PEG fusion protocol with MB diluted in PlasmaLyte-A supplemented with EGTA, no evoked electrical response was elicited after completing the protocol with MB diluted in diH_2_O lacking EGTA. While it is possible that PEG fusion in rodents may not require EGTA, our data suggest that EGTA may be necessary in all steps before PEG application in a porcine facial nerve injury model. Although the exact reason remains unknown, we have two potential hypotheses related to the calcium chelating agent: (1) the porcine facial nerve might be more sensitive to calcium ions compared to the rat sciatic nerve and (2) more calcium ions may be present within the surgical site compared to the rat surgical field. A third hypothesis might be that the dilutions have different osmolarity, which may be an important consideration for successful PEG fusion (26). While we found evidence of successful PEG fusion in a porcine model using Plasmalyte with EGTA, hypotonic 1% MB diluted in Plasmalyte with EGTA, followed by PEG application and finally lactated ringers' solution, a limitation of this study is the low sample size. Therefore, future studies with larger sample sizes should be conducted to determine the mechanisms underlying this discrepancy, as well as the relevance to surgical repair of nerves in humans.

Surgical technique is also likely an important factor for reproducible outcomes in this paradigm. Although we employed standard microsurgical repair techniques, it is important to ensure that suturing resulted in proper fascicular alignment with minimal tension and/or bunching at the repair site. Indeed, a previous study has reported that 67% of surgeons trained to perform neurorrhaphy achieved successful “fusion” after practice (with success defined based on restoring acute electrophysiological conduction and subsequent behavioral recovery) (10). Thus, ensuing optimal surgical repair techniques and experience with the PEG protocol are important considerations when executing nerve fusion experiments.

As innovative neurosurgical repair strategies and advanced regenerative therapies are further progressed, there will be an increased need for clinically relevant models to optimize these strategies prior to clinical implementation. Most preclinical research in peripheral nerve repair and regeneration has focused on rodent models (12, 27, 28). Nevertheless, rodent experiments of peripheral nerve injury do not effectively replicate the major impediments to recovery encountered in the clinical setting, such as large diameter nerve injury and long total regenerative distances to reach distal targets (13, 29). In addition, it is unclear to what extent rodent nerve regeneration recapitulates human physiological processes. While rodent models serve a necessary purpose of initial proof-of-concept studies, true translational experiments of peripheral nerve injury and repair call for large animal models to effectively model human injury responses (11).

The porcine facial nerve model offers several advantages that may be useful for future studies assessing the long-term efficacy and functional recovery of PEG fusion. Access to the facial nerve is straightforward, requiring only minimal dissection. The anatomy of the nerve is predictable and easy to identify. Damage to the nerve results in minimal morbidity and can be performed bilaterally. The facial nerve does not cross a joint, so any nerve repair experimentation is not at risk of mechanical disruption, unlike long nerve repairs in the hind limb. The regenerative distance to the snout is a fraction of the distance in the hind limb, and can facilitate more rapid results even following long-gap repairs. Many of the indirect techniques used to assess nerve regeneration and functional recovery, such as ultrasonography and electrophysiology, can be performed with greater ease and access. For direct stimulation, the nerve repair can be easily re-exposed with minimal morbidity. Conversely, because of the conserved position of the nerve just under the skin, surface electrode placement can be carried out with high reliability. Notably, ~4–5 cm of nerve can be easily removed, allowing for testing of long gap nerve repairs with relatively close proximity to the end target. Previous studies have reported PEG fusion following short gap autograft repair (20); future studies in large animals may include testing PEG fusion following long gap autograft repair.

While our proof-of-concept study demonstrates PEG fusion may enable early electrophysiological recovery with potentially corroborating histological evidence of axons within the graft region, further investigation is necessary to determine whether PEG fusion following nerve repair in large animals results in permanently fused axons and/or mitigates Wallerian degeneration. Although PEG is a commonly utilized compound with tunable properties that has been shown to be safe in humans for various applications (30), the impact of high molecular weight formulation on long-term physiology has yet to be studied. Therefore, future large animal safety and efficacy studies should assess whether there are any adverse long-term effects of PEG application and/or undesirable effects on nerve function.

## Conclusion

Several promising strategies have been proposed for the treatment of severe nerve injury, such as PEG-mediated axon fusion for acute nerve repair. For most strategies, mechanistic efficacy testing in large animal models will be advantageous prior to clinical deployment. Here, we identified a PEG fusion protocol that enabled the rapid restoration of electrophysiological connection immediately after neurorrhaphy, with putative axons bridging the transection site, in a porcine model of facial nerve injury. The porcine model of facial nerve injury presented here is suitable for testing novel nerve repair strategies by adequately representing major challenges experienced in the clinical setting.

## Data Availability Statement

The raw data supporting the conclusions of this article will be made available by the authors upon reasonable request.

## Ethics Statement

The animal study was reviewed and approved by University of Pennsylvania's Institutional Animal Care and Use Committee.

## Author Contributions

DC, DP, and JB conceived and designed the experiments, and completed the initial figure and manuscript preparation. DP and JB established the surgical approach and electrophysiological benchmarks. DP performed all neurorrhaphy procedures. JB conducted the electrophysiology assessments. KB and SR assisted with surgical implementation, reagent preparation, and/or electrophysiological testing. ZA assisted with surgical implementation and model development. JB and FL performed histological assessments, confocal imaging, data analyses, and figure preparation. DC oversaw all studies and manuscript preparation. All authors provided edits and comments to the manuscript.

## Funding

Financial support provided by the U.S. Department of Defense [CDMRP/JPC8 – CRMRP #W81XWH-16-1-0796 (DC) and MRMC #W81XWH-15-1-0466 (DC)], the National Institutes of Health [R43-NS108869 (DC)], the Department of Neurosurgery at the University of Pennsylvania (DP and DC), and the American Association of Neurological Surgeons and Congress of Neurological Surgeons [Codman Fellowship in Neurotrauma and Critical Care (DP)].

## Conflict of Interest

The authors declare that the research was conducted in the absence of any commercial or financial relationships that could be construed as a potential conflict of interest.

## Publisher's Note

All claims expressed in this article are solely those of the authors and do not necessarily represent those of their affiliated organizations, or those of the publisher, the editors and the reviewers. Any product that may be evaluated in this article, or claim that may be made by its manufacturer, is not guaranteed or endorsed by the publisher.

## References

[1] Spencer MR Irving MM. Causes and management of facial nerve palsy. Br J Hosp Med. (2016) 77:686–91. 10.12968/hmed.2016.77.12.686 27937022

[2] Gordin E Lee TS Ducic Y Arnaoutakis D. Facial nerve trauma: evaluation and considerations in management. Craniomaxillofac Trauma Reconstr. (2015) 8:1–13. 10.1055/s-0034-1372522 25709748 PMC4329040

[3] Fu SY Gordon T. Contributing factors to poor functional recovery after delayed nerve repair: prolonged denervation. J Neurosci. (1995) 15:3886–95. 10.1523/JNEUROSCI.15-05-03886.1995 7751953 PMC6578254

[4] Fu SY Gordon T. Contributing factors to poor functional recovery after delayed nerve repair: prolonged axotomy. J Neurosci. (1995) 15:3876–85. 10.1523/JNEUROSCI.15-05-03876.1995 7751952 PMC6578210

[5] Ghergherehchi CL Bittner GD Hastings RL Mikesh M Riley DC Trevino RC. Effects of extracellular calcium and surgical techniques on restoration of axonal continuity by polyethylene glycol fusion following complete cut or crush severance of rat sciatic nerves. J Neurosci Res. (2016) 94:231–45. 10.1002/jnr.23704 26728662 PMC4715960

[6] Pfister BJ Gordon T Loverde JR Kochar AS Mackinnon SE Cullen DK. Biomedical engineering strategies for peripheral nerve repair: surgical applications, state of the art, and future challenges. Crit Rev Biomed Eng. (2011) 39:81–124. 10.1615/CritRevBiomedEng.v39.i2.20 21488817

[7] Riley DC Bittner GD Mikesh M Cardwell NL Pollins AC Ghergherehchi CL. Polyethylene glycol-fused allografts produce rapid behavioral recovery after ablation of sciatic nerve segments. J Neurosci Res. (2015) 93:572–83. 10.1002/jnr.23514 25425242 PMC4329031

[8] Bittner GD Sengelaub DR Trevino RC Ghergherehchi CL Mikesh M. Robinson and madison have published no data on whether polyethylene glycol fusion repair prevents reinnervation accuracy in rat peripheral nerve. J Neurosci Res. (2017) 95:863–6. 10.1002/jnr.23849 27514994 PMC5241247

[9] Mikesh M Ghergherehchi CL Hastings RL Ali A Rahesh S Jagannath K. Polyethylene glycol solutions rapidly restore and maintain axonal continuity, neuromuscular structures, and behaviors lost after sciatic nerve transections in female rats. J Neurosci Res. (2018) 96:1223–42. 10.1002/jnr.24225 29659058 PMC5980706

[10] Ghergherehchi CL Mikesh M Sengelaub DR Jackson DM Smith T Nguyen J. Polyethylene glycol (PEG) and other bioactive solutions with neurorrhaphy for rapid and dramatic repair of peripheral nerve lesions by PEG-fusion. J Neurosci Methods. (2019) 314:1–12. 10.1016/j.jneumeth.2018.12.015 30586569 PMC6475191

[11] Kaplan HM Mishra P Kohn J. The overwhelming use of rat models in nerve regeneration research may compromise designs of nerve guidance conduits for humans. J Mater Sci Mater Med. (2015) 26:226. 10.1007/s10856-015-5558-4 26296419 PMC4545171

[12] Angius D Wang H Spinner RJ Gutierrez-Cotto Y Yaszemski MJ Windebank AJ. systematic review of animal models used to study nerve regeneration in tissue-engineered scaffolds. Biomaterials. (2012) 33:8034–9. 10.1016/j.biomaterials.2012.07.056 22889485 PMC3472515

[13] Burrell JC Browne KD Dutton JL Das S Brown DP Laimo FA. A Porcine model of peripheral nerve injury enabling ultra-long regenerative distances: surgical approach, recovery kinetics, and clinical relevance. bioRxiv. (2019) 2019:610147. 10.1101/610147 32392341

[14] Siemionow M Brzezicki G. Chapter 8: Current techniques and concepts in peripheral nerve repair. Int Rev Neurobiol. (2009) 87:141–72. 10.1016/S0074-7742(09)87008-6 19682637

[15] Robinson L.R. Traumatic injury to peripheral nerves. Muscle Nerve. (2000) 23:863–73. 10.1002/(SICI)1097-4598(200006)23:6<863::AID-MUS4>3.0.CO;2-0 10842261

[16] Brattain K. Analysis of the Peripheral Nerve Repair Market in the United States. Minneapolis, MN: Magellan Medical Technology Consultants, Inc. (2012).

[17] Bittner GD Ballinger ML Raymond MA. Reconnection of severed nerve axons with polyethylene glycol. Brain Res. (1986) 367:351–5. 10.1016/0006-8993(86)91617-3 3697710

[18] Krause TL Bittner GD. Rapid morphological fusion of severed myelinated axons by polyethylene glycol. Proc Natl Acad Sci USA. (1990) 87:1471–5. 10.1073/pnas.87.4.1471 2304913 PMC53497

[19] Bittner GD Rokkappanavar KK Peduzzi JD. Application and implications of polyethylene glycol-fusion as a novel technology to repair injured spinal cords. Neural Regen Res. (2015) 10:1406–8. 10.4103/1673-5374.162772 26604897 PMC4625502

[20] Sexton KW Pollins AC Cardwell NL Del Corral GA Bittner GD Shack RB. Hydrophilic polymers enhance early functional outcomes after nerve autografting. J Surg Res. (2012) 177:392–400. 10.1016/j.jss.2012.03.049 22521220 PMC4096106

[21] Bittner GD Keating CP Kane JR Britt JM Spaeth CS Fan JD. Rapid, effective, and long-lasting behavioral recovery produced by microsutures, methylene blue, and polyethylene glycol after completely cutting rat sciatic nerves. J Neurosci Res. (2012) 90:967–80. 10.1002/jnr.23023 22302646

[22] Britt JM Kane JR Spaeth CS Zuzek A Robinson GL Gbanaglo MY. Polyethylene glycol rapidly restores axonal integrity and improves the rate of motor behavior recovery after sciatic nerve crush injury. J Neurophysiol. (2010) 104:695–703. 10.1152/jn.01051.2009 20445038

[23] Yoo S Nguyen MP Fukuda M Bittner GD Fishman HM. Plasmalemmal sealing of transected mammalian neurites is a gradual process mediated by Ca(2+)-regulated proteins. J Neurosci Res. (2003) 74:541–51. 10.1002/jnr.10771 14598298

[24] Fishman HM Bittner GD. Vesicle-mediated restoration of a plasmalemmal barrier in severed axons. News Physiol Sci. (2003) 18:115–8. 10.1152/nips.01429.2002 12750447

[25] Krause TL Marquis RE Lyckman AW Ballinger ML Bittner GD. Rapid artificial restoration of electrical continuity across a crush lesion of a giant axon. Brain Res. (1991) 561:350–3. 10.1016/0006-8993(91)91615-8 1802349

[26] Vestad B Llorente A Neurauter A Phuyal S Kierulf B Kierulf P. Size and concentration analyses of extracellular vesicles by nanoparticle tracking analysis: a variation study. J Extracell Vesicles. (2017) 6:1344087. 10.1080/20013078.2017.1344087 28804597 PMC5533132

[27] Kemp SW Cederna PS Midha R. Comparative outcome measures in peripheral regeneration studies. Exp Neurol. (2017) 287:348–57. 10.1016/j.expneurol.2016.04.011 27094121

[28] Navarro X. Functional evaluation of peripheral nerve regeneration and target reinnervation in animal models: a critical overview. Eur J Neurosci. (2016) 43:271–86. 10.1111/ejn.13033 26228942

[29] Burrell JC Bhatnagar D Brown DP Murthy NS Dutton J Browne KD. Tyrosine-derived polycarbonate nerve guidance tubes elicit proregenerative extracellular matrix deposition when used to bridge segmental nerve defects in swine. J Biomed Mater Res A. (2020) 11:902007. 10.1101/2020.01.11.902007 32985789

[30] D'Souza A A Shegokar R. Polyethylene glycol (PEG): a versatile polymer for pharmaceutical applications. Expert Opin Drug Deliv. (2016) 13:1257–75. 10.1080/17425247.2016.1182485 27116988

[31] Ghergherehchi CL Shores JT Alderete J Weitzel EK Bittner GD. Methylene blue enhances polyethylene glycol-fusion repair of completely severed rat sciatic nerves. Neural Regen Res. (2021) 16:2056–63. 10.4103/1673-5374.308099 33642394 PMC8343334

